# Nordic inflammatory bowel disease treatment strategy trial: protocol for the NORDTREAT randomised controlled biomarker-strategy trial

**DOI:** 10.1136/bmjopen-2023-083163

**Published:** 2024-07-31

**Authors:** Martin Rejler, Johannes David Füchtbauer, Lóa G Davíðsdóttir, Anja Fejrskov, Johan D Söderholm, Robin Christensen, Vibeke Andersen, Dirk Repsilber, Jens Kjeldsen, Marte Høivik, Jonas Halfvarson

**Affiliations:** 1 School of Health and Medical Sciences, Örebro University, Örebro, Sweden; 2 Futurum Academy of Health and Care, Jönköping, Sweden; 3 Jönköping Academy for Improvement in Health and Welfare, Jönköping University, Jönköping, Sweden; 4 Internal Medicine & Emergency Department, Odense University Hospital, Svendborg, Denmark; 5 Research Unit of Medical Gastroenterology, Department of Clinical Research, University of Southern Denmark, Odense, Denmark; 6 Department of Medical Gastroenterology, Odense University Hospital, Odense, Denmark; 7 Department of Gastroenterology, Landspitali National University Hospital of Iceland, Reykjavik, Iceland; 8 Molecular Diagnostics and Clinical Research Unit, Institute of Regional Health Research, University Hospital of Southern Denmark, Hospital Sønderjylland, Aabenraa, Denmark; 9 Section for Biostatistics and Evidence-Based Research, the Parker Institute, Bispebjerg and Frederiksberg Hospital, Copenhagen, Denmark; 10 Department of Biomedical and Clinical Sciences, Linköping University, Linköping, Sweden; 11 Research Unit of Rheumatology, Department of Clinical Research, University of Southern Denmark, Odense University Hospital, Odense, Denmark; 12 Institute of Regional Research, University of Southern Denmark, Odense, Denmark; 13 Institute of Molecular Medicine, University of Southern Denmark, Odense, Denmark; 14 OPEN - Open Patient Data Explorative Network, University of Southern Denmark, Odense, Denmark; 15 Department of Gastroenterology, Oslo University Hospital, Oslo, Norway; 16 Institute of Clinical Medicine, University of Oslo, Oslo, Norway; 17 Department of Gastroenterology, Faculty of Medicine and Health, Örebro University, Örebro, Sweden

**Keywords:** inflammatory bowel disease, randomised controlled trial, prognosis

## Abstract

**ABSTRACT:**

**Introduction:**

The absence of reliable prognostic markers poses a challenge to the management of inflammatory bowel disease (IBD). Patients with aggressive disease may not receive sufficient treatment with conventional ‘step-up’ therapy, whereas a top-down approach may expose patients with indolent disease to unnecessary treatment-related toxicity. The objective of the Nordic IBD treatment strategy trial (NORDTREAT) is to assess the feasibility of personalised therapy by stratifying patients according to a prognostic serum protein signature at diagnosis.

**Methods and analysis:**

NORDTREAT is a multicentre, biomarker-strategy design, open-label controlled trial. After screening consent, eligible patients are randomised (1:1) into one of two groups: a group with access to the protein signature and a group without access. In the access to protein signature group, patients displaying a protein signature suggestive of an increased risk of an aggressive disease course will be treated in line with a top-down treatment algorithm (anti-tumour necrosis factor agent with/without an immunomodulator). In contrast, those with a protein signature indicative of indolent disease will be excluded from the trial. Patients not in the access group receive treatment based on clinical management. This traditional management involves a stepwise escalation of treatment as determined by the investigator after failure of first-line treatment. After 52 weeks, outcomes are assessed in the subgroup of patients with a protein profile indicating a potentially severe disease trajectory. The primary endpoint is a composite of the proportion of patients with corticosteroid-free clinical and endoscopic remission at week 52. Surgical intervention due to IBD during follow-up will be defined as treatment failure.

**Ethics and dissemination:**

Ethical approval has been obtained, and recruitment is underway at sites in four participating Nordic countries (Denmark, Iceland, Norway and Sweden). Following trial completion and data analysis, the trial results will be submitted for publication in peer-reviewed journals and presented at international conferences.

**Trial registration number:**

NCT05180175; Pre-results. EudraCT number: 2019-002942-19.

**STRENGTHS AND LIMITATIONS OF THIS STUDY**

- This study is the first multicentre randomised biomarker-strategy design trial comparing the outcome of top-down versus clinical management in a biomarker-defined subgroup of patients with newly diagnosed Crohn’s disease and ulcerative colitis.
- Results can potentially demonstrate that personalised therapy can be effectively delivered to patients diagnosed with Crohn’s disease and ulcerative colitis using a prognostic serum protein signature.
- Only patients with a predicted increased risk of severe disease progression, as defined by the protein signature, will be compared.
- Initiation of top-down therapy is defined as the start of an anti-tumour necrosis factor agent and an immunomodulator, and it does not consider other advanced treatments.
- Neither participants nor treating physicians are blinded to treatment.

## Introduction

### Background

 Inflammatory bowel disease (IBD) is a chronic progressive disease characterised by inflammation in the gastrointestinal tract. Its prevalence has steadily increased, impacting approximately 0.5%–1% of the Nordic population. Crohn’s disease, ulcerative colitis and IBD-unclassified represent the three subtypes of IBD.^1^,^4^ Symptoms of IBD include diarrhoea, rectal bleeding, abdominal pain, fatigue and unintended weight loss. As such, the disease significantly affects the patient’s quality of life. There is considerable heterogeneity among patients with IBD, with significant variability in disease progression.^5^

Current IBD treatments are expensive and lack therapeutic precision, resulting in reduced efficacy, safety concerns and increased risk of disease progression. According to prevailing standards of care, patients are often treated using a ‘step-up’ approach, starting with corticosteroids or 5-aminosalicylates and escalating stepwise to more advanced therapies in cases of insufficient response or recurrent flares. This strategy aims to not overtreat patients, but it will unavoidably result in disease progression in some patients while they are insufficiently treated. Moreover, fragmented healthcare, limited understanding of a patient’s disease history and restricted access to clinical expertise often cause delays in treatment adaptation, excessive use of corticosteroids and increased risk of disease complications and need for surgery.

Over the past two decades, several targeted therapies, including various biological agents, have been approved for the treatment of IBD.^6^ Growing evidence shows that early introduction of these drugs improves clinical outcomes. The benefit of early initiation of potent drugs has primarily been shown for anti-tumour necrosis factor (TNF) agents. In 2008, investigators of ‘the Step-Up vs Top-Down Trial’ demonstrated that the early introduction of infliximab was superior to conventional step-up treatment.^7^ However, administering anti-TNF therapy to all IBD patients at an early stage would expose those with a potentially mild disease progression to unnecessary risks of treatment side effects and would incur additional expenses for medication.

Alternative approaches to address this clinical dilemma have been explored. In open-label cluster randomised controlled trials, such as the REACT I and II studies, early enhanced care algorithms have been compared with conventional management in patients with Crohn’s disease.^8^ However, the absence of a difference between treatment algorithms in these trials illustrates the pressing need to identify reliable predictors of patients who are at increased risk of progressing and developing severe disease with complications and who would benefit from early aggressive treatment with targeted drugs such as anti-TNF agents. After the identification of a gene expression signature in peripheral blood CD8+ T cells that correlated with the future progression of Crohn’s disease,^9^,^11^ Biasci *et al* developed a whole blood quantitative polymerase chain reaction (PCR) assay capable of analysing this signature without requiring cell separation.^12^ The prognostic utility of this assay was explored in the recently reported trial: Predicting outcomes for Crohn’s disease using a molecular biomarker (PROFILE).^13^

As a partner of the IBD Character Consortium, we recently identified a prognostic serum protein signature for early IBD.^14^ Using proximity extension assay methodology and relative quantification of 460 proteins, we identified 12 proteins independently associated with treatment escalation in early IBD. The signature allowed us to differentiate patients with an aggressive disease course (defined as the need for biologics/cyclosporine or surgery after initial disease remission) from those with a mild disease. We recently identified additional proteins associated with a future severe disease progression within the Swedish inception cohort in IBD.^15^ Combining the prognostic proteins from these two cohorts holds promise for early risk stratification and individualised treatment approaches in IBD. To facilitate the translation of this combined signature into a clinical trial setting, we have, together with Olink Proteomics, Uppsala, Sweden, developed a custom-plex panel capable of measuring the absolute concentrations of the 13 proteins that contribute to the combined signature (manuscript in preparation). Our next objective is to conduct a randomised controlled biomarker-stratified trial to investigate whether this protein signature can facilitate the delivery of personalised medicine in IBD, ultimately leading to improved outcomes in patients with a higher likelihood of experiencing a severe disease trajectory. This trial will help determine the clinical utility of the prognostic protein signature and its potential to guide tailored treatment strategies in patients with newly diagnosed IBD.

This manuscript summarises the approved Nordic IBD treatment strategy trial (NORDTREAT) protocol used at publication (V.1.4, 5 October 2023). If any protocol or patient information sheet changes, the approval of the relevant medical product agencies and the ethics committees will be required, as applicable.

### Hypothesis, aims and objectives

Our hypothesis suggests that stratifying patients based on a biomarker-driven assessment of their future disease progression could enhance treatment efficacy, optimise clinical care and support the integration of personalised medicine in IBD. To achieve this, a possible approach would involve identifying and treating patients expected to develop a more aggressive disease course using top-down therapy. Simultaneously, it would be essential to safeguard individuals likely to experience a less severe disease from the potential risks associated with the early introduction of unnecessary immunosuppression.

Therefore, the NORDTREAT trial aims to evaluate whether access to a prognostic protein signature at the initial diagnosis of IBD, coupled with the use of top-down therapy with an anti-TNF agent in high-risk patients identified by the signature, can improve treatment outcomes in this subset of patients. Also, the trial will assess whether this treatment strategy is safe and can improve these patients’ quality of life and health resource allocation. The anticipated results of this study hold the potential to showcase the efficacy of administering personalised therapy to patients diagnosed with Crohn’s disease and ulcerative colitis using a prognostic serum protein signature.

The primary aim is to assess the impact of top-down treatment compared with clinical management on the proportion of patients achieving corticosteroid-free clinical and endoscopic remission by week 52 in a biomarker-defined subgroup of patients with predicted severe disease course. Surgery because of IBD during follow-up will be defined as treatment failure.

The key secondary aims are to compare the effect of top-down treatment versus clinical management on clinical remission, endoscopic remission, clinical response, endoscopic response and drug-related adverse events (AEs) in a biomarker-defined subgroup of patients with predicted severe disease course.

Other secondary aims include, time to occurrence of the first major adverse outcome, the cumulative glucocorticoid use over time through week 52, the change from baseline in C-reactive protein (CRP) concentration over time through week 52, the change from baseline in faecal calprotectin concentration over time through week 52, the change from baseline in each of the four dimensions of the IBD Questionnaire (IBDQ) at 52 weeks, the proportion of patients with >20-point improvement from baseline in the IBDQ score at 52 weeks, the change from baseline for the 36-item Short Form Health Survey (SF-36), including an algorithm yielding two summary scores, the Physical Component Summary and the Mental Component Summary scores at 52 weeks, the changes from baseline in the EuroQoL-5 Dimensions-5 Levels (EQ-5D-5L) Health Questionnaire and the health state visual analogue scale (EQ-VAS) scores at 52 weeks and maintenance of clinical remission through week 52 in patients who achieved clinical remission at week 12.

## Methods and analysis

### Study design

**Figure 1:**
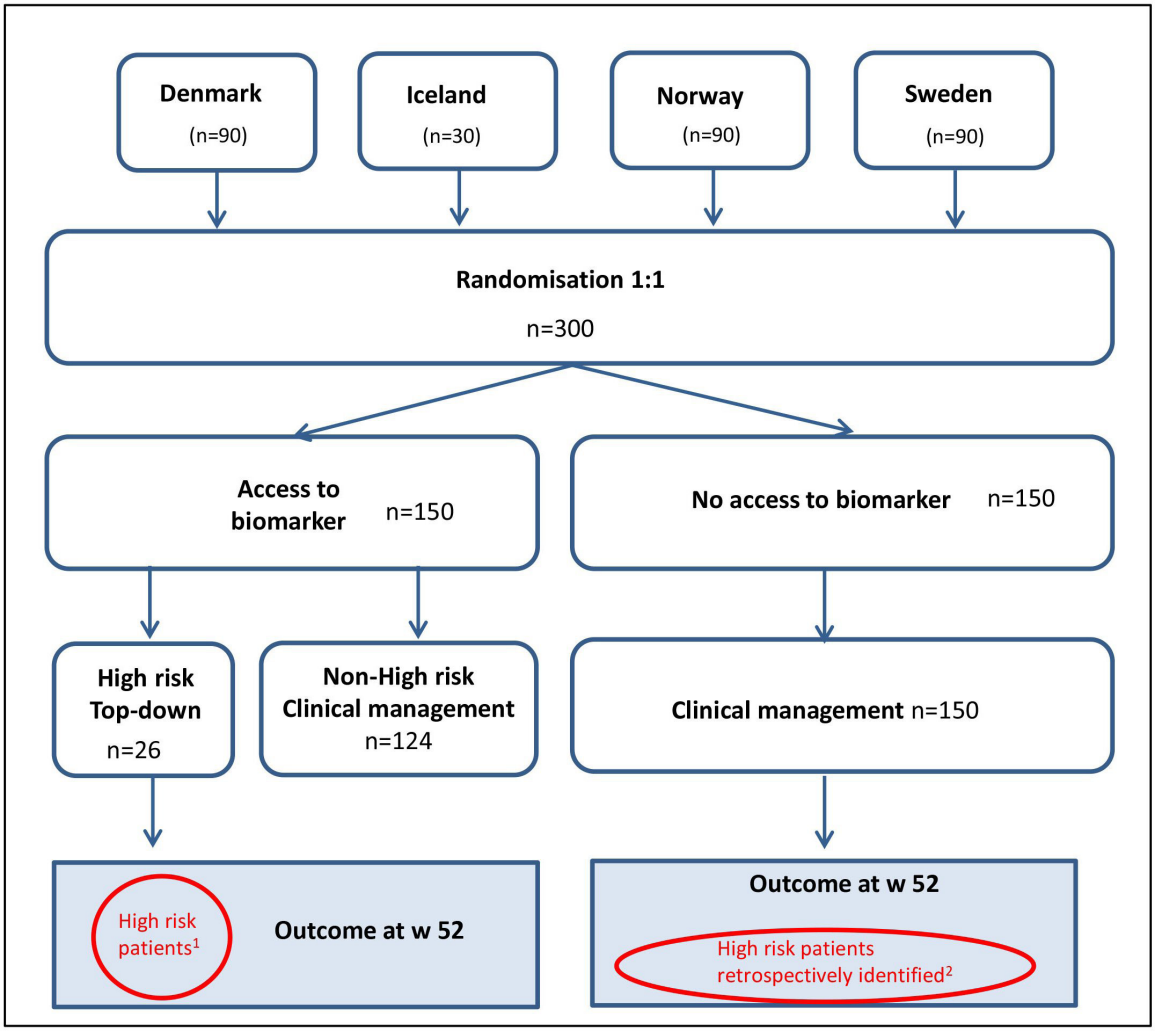
Trial design of the Nordic IBD treatment strategy trial (NORDTREAT). After undergoing screening and consent, eligible patients are assigned at random in a 1:1 ratio to either a group with access to the protein signature or a group without access to the protein signature. Patients in the protein signature arm with a ‘high-risk’ protein profile are treated in line with a top-down algorithm. In contrast, patients in the arm without access to the protein signature receive treatment based on clinical management, that is, a gradual intensification of treatment as determined by the investigator. ^1^Estimated proportion meeting the 1’ endpoint 75%. ^2^Estimated proportion meeting the 1’ endpoint 35%.

NORDTREAT is a multicentre, biomarker-stratified, open-label controlled trial. After screening and consent (online supplemental material), eligible patients are randomised (1:1) to a group with or without access to the protein signature (figure 1). Patients assigned to the access to protein signature arm who display a protein profile associated with heightened susceptibility to a severe disease trajectory will receive treatment based on a top-down treatment algorithm involving an anti-TNF agent with/without an immunomodulator. In contrast, those with a protein signature indicative of a slow disease progression will be excluded from the trial. Patients assigned to the group lacking access to the protein signature will receive treatment based on standard clinical management. Patients who do not respond to first-line treatment will have their treatment escalated stepwise, as determined by the investigator. The use of therapeutic drug monitoring for treatment optimisation will be permitted. Study participants with a protein profile predictive of poor disease course, who have been treated according to the protein signature-based top-down approach, are compared with the corresponding group treated according to conventional clinical management who subsequently, after completion of the 52-week trial, are found to have a protein profile predictive of poor disease course at baseline.

As part of the NORDTREAT initiative, the prospective NORDTREAT cohort study is also conducted in parallel with the randomised NORDTREAT intervention trial.^16^ The cohort study seeks to identify novel molecular biomarkers of diagnostic and prognostic value.

### Trial sites, duration and visits

NORDTREAT is a multicentre clinical trial conducted across four Nordic countries. At publication, 15 sites were initiated in Denmark, Iceland, Norway and Sweden. After completing the informed consent process, patients will undergo screening and be enrolled at baseline. The trial will then continue for 52 weeks with regular follow-up. There will be three visits throughout the study period, during which data are collected and recorded. The study visits are scheduled for 12, 26 and 52 weeks after baseline. In February 2022, the trial started with the enrolment of the first patient. The final visit of the last patient defines the end of the trial.

### Eligibility criteria

Patients who satisfy all the inclusion criteria and do not meet any exclusion criteria will qualify for enrolment. Box 1 contains a complete list of the eligibility criteria. The target population is incident treatment-naïve patients with newly diagnosed IBD based on clinical history and examination, negative stool cultures and macroscopic appearance of IBD at endoscopy or imaging.^17^ However, the histopathology report from the endoscopy is not mandatory to confirm eligibility and inclusion in the study. If clinically relevant, rescreening is allowed. Patients with a previous diagnosis of IBD (before the current episode) are not eligible for inclusion, as outlined in the first exclusion criteria.

Box 1 **Eligibility criteria for the Nordic inflammatory bowel disease treatment strategy trial (NORDTREAT)**

**Inclusion criteria**

Patients must fulfil all the criteria listed below to be included in the study.

- Ulcerative colitis (UC) or Crohn’s disease (CD) diagnosed within <4 weeks using standard endoscopic, histologic or radiological criteria. Histology reports may not be available at baseline.
- Naïve to immunomodulators, biologics and small molecules, that is Janus kinas (JAK) inhibitors.
- Aged 18–70 years.
- Considered eligible according to tuberculosis screening criteria.
- Provide written informed consent to participate in the study.

**Exclusion criteria**

The presence of any exclusion criteria precludes inclusion.

- A previous known diagnosis of CD, ulcerative colitis or inflammatory bowel disease-unclassified >6 weeks before baseline.
- Unable to provide informed consent.
- Not able to comply with protocol requirements (eg, for reasons including alcohol or recreational drug abuse).
- Ongoing sepsis.
- Acute obstructive symptoms and evidence of a fixed stricture on radiology or colonoscopy suggest that the patient needs surgery over the following year. Based on the clinician’s judgement, patients with modest degrees of strictures on imaging but no obstructive symptoms may be included.
- Contraindications to trial medications, including a history of hepatitis B or C, tuberculosis, cardiac failure, the New York Heart Association (NYHA) III–IV or hypersensitivity. Hypersensitivity to a thiopurine agent should alert the prescriber to probable hypersensitivity to other thiopurines.
- History of malignancy.
- Pregnancy.*
- Other serious medical or psychiatric illness.

* pPregnancy test (urine) should be done at the baseline visit for female participants not in menopause, as this is important for planned diagnostic procedures, including endoscopy and radiological procedures.

### Patient and public involvement

The development and advancement of individualised medicine in IBD constitute a significant goal for patients, healthcare and society. Patients have been represented and advised on the development of the protocol through the Patient Advisory Council (PAC), with representatives from the Nordic countries. Members of the PAC are also involved in the recruitment and conduct of the NORDTREAT trial. After the trial is completed and reported, the trial results will be distributed to all trial participants and patient organisations in the Nordic countries. The general public will also be informed through press releases and public engagement activities, which will be organised in partnership with hospitals, universities and the PAC.

### Outcome measures

The Mayo Score will serve as the primary measure for assessing disease activity response to treatment in patients with ulcerative colitis. Clinical disease activity will be evaluated based on patient-reported outcomes from the per-adapted Mayo Score, that is, average daily absolute stool number and rectal bleeding subscore. Endoscopic activity will be evaluated based on the endoscopic Mayo subscore. The primary method instrument for assessing treatment response in patients with Crohn’s disease will be based on the average daily stool frequency, average daily abdominal pain (generated from the Crohn’s Disease Activity Index) and endoscopic activity determined by the Short Endoscopic Score for Crohn’s Disease. The degree of inflammation for ulcerative colitis and Crohn’s disease will be assessed by measuring serum CRP concentrations and faecal calprotectin. Patient well-being will be evaluated using the IBDQ, the SF-36 and the EQ-5D. All primary and secondary endpoints are listed in box 2.

Box 2 **Primary and secondary endpoints and outcomes in the Nordic IBD treatment strategy trial (NORDTREAT)**

**Primary endpoint**

As defined below, a composite outcome of corticosteroid-free*, clinical remission and endoscopic remission at week 52. Surgery because of IBD during follow-up will be defined as treatment failure.

Ulcerative colitis:

- Clinical remission per patient reported Mayo: a stool frequency subscore (SFS) ≤1, and not greater than baseline, and a Rectal Bleeding Subscore (RBS) of 0.
- Endoscopic remission: an endoscopic Mayo subscore of 0 (or in patients without endoscopy at week 52, normalisation of f-calprotectin, defined as <250 µg/g (EK-Cal, Thermo Fisher Scientific, Uppsala Sweden)).

Crohn’s disease:

- Clinical remission: an average daily stool frequency (SF) of ≤2.8 and not worse than baseline and an average daily abdominal pain (AP) score of ≤1 and not worse than baseline.
- Endoscopic remission: Short Endoscopic Score for Crohn’s Disease ≤2 (or in patients without endoscopy at week 52, normalisation of f-calprotectin, defined as <250 µg/g (EK-Cal, Thermo Fisher Scientific, Uppsala Sweden)).

**Key secondary endpoints**

1. Proportion of participants with clinical remission at 52 weeks.
2. Proportion of participants with endoscopic remission at 52 weeks.
3. Proportion of participants with a clinical response:
  - Ulcerative colitis: a decrease from baseline in the adapted Mayo Score (range 0–9, with higher scores indicating more severe disease) by ≥30% or ≥2 points, with either a decrease from baseline in the RBS of ≥1 or an absolute RBS of ≤1.
  - Crohn’s disease: ≥30% decrease in average daily SF or ≥0% decrease in the average daily AP score, where both are not worse than baseline.
4. Proportion of participants with an endoscopic response:
  - Ulcerative colitis: an endoscopic Mayo subscore of ≤1 (or in patients without endoscopy at week 52, a reduction of f-calprotectin by ≥50% compared with baseline).
  - Crohn’s disease: decrease in SES-CD >50% from baseline (or for a baseline SES-CD of 4, at least a 2-point reduction from baseline) (or in patients without endoscopy at week 52, a decrease of faecal calprotectin by ≥50% compared with baseline).
5. The proportion of patients with drug-related AEs.

**Other secondary outcomes**

1. Time to occurrence of the first major adverse outcome, defined as the composite of surgery or hospital admission for IBD or development of a serious disease-related complication (the individual components of this outcome will also be assessed independently). Serious complications include the occurrence of substantially worsening disease activity defined by:
  - New abscess, fistula or stricture among Crohn’s disease patients.
  - Progression in disease extent among ulcerative colitis patients.
  - Extra-intestinal manifestations among patients with Crohn’s disease or ulcerative colitis.
2. Cumulative glucocorticoid (measured as prednisolone equivalents) over 52 weeks.
3. Change from baseline in C-reactive protein (CRP) concentration through week 52.
4. Change from baseline in faecal calprotectin concentration through week 52.
5. Change from baseline in each of the four dimensions of the IBDQ at 52 weeks.
6. Proportion of participants with >20-point improvement from baseline in the IBDQ score at 52 weeks.
7. Change from baseline for each of the eight individual subscales of the 36-item Short Form Health Survey and the Physical Component Summary and Mental Component Summary scores at 52 weeks.
8. Changes from baseline in the EuroQoL-5 Dimensions (EQ-5D), EQ-5D index and EQ-visual analogue scale scores at 52 weeks.
9. Proportion of participants with sustained clinical remission at week 52 out of those who had initially achieved clinical remission at week 12.
10. Proportion of participants with normalisation of faecal calprotectin concentration: faecal calprotectin concentration <250 mg/kg (above defined as <250 µg/g).
11. Cumulative use of healthcare resources, defined as surgical procedures, number of days of hospitalisation, healthcare visits to nurses, dieticians and doctors, imaging procedures, endoscopies, use of IBD-associated treatments and corresponding costs through week 52.

*Corticosteroid-free: no oral, parenteral or topical corticosteroid use within the past 4 weeks.

### Health economic evaluation

The Swedish Institute for Health Economics will conduct a health economic evaluation on the cumulative use of healthcare resources up to week 52. Healthcare resources encompass IBD-related surgical procedures, hospitalisations, healthcare visits to nurses, dieticians and doctors, use of imaging procedures and endoscopies, as well as treatments and their corresponding costs.

### Adverse events

Per the mandatory reporting requirements, we will collect and disclose the number of withdrawals resulting from AEs, mortality during the 52- week observation period and the number of patients experiencing one or more serious AEs within each group. During each study visit, the investigator will ask open-ended questions about any AEs the participants may have encountered since their last visit. The investigator will determine whether a causal relationship exists between an AE and the use of the investigational product, categorising them as likely related, possibly related or unrelated. A Data Safety Monitoring Board will monitor clinical outcome data and AEs to ensure the continuing safety of the participants enrolled in the study. Safety reports will be submitted to the regulatory authorities and ethics committees in compliance with each participating country’s requirements.

### Study procedures and assessments

Patients referred for suspicion of IBD and those with newly diagnosed IBD (within 4 weeks of diagnosis) will be identified by local clinical team members and prescreened for participation in the NORDTREAT study. Potential trial patients will be allocated a study identifier at screening through the electronic case report form (eCRF). Before inclusion in the study and randomisation, patients must satisfy all inclusion criteria and none of the exclusion criteria.

Study data registered by clinicians, study nurses and technicians will be stored in a web-based CRF (Viedoc, Uppsala, Sweden). Participants will access the questionnaires via the investigator’s electronic link on MyViedoc or paper. All data will be stored in secure research storage facilities. To guarantee the trial’s integrity and quality, including complete follow-up visits, a network of independent Nordic monitors will be tasked with responsibilities such as site initiations, audits, data verification, compliance checks and close-out visit management.

### Randomisation

At baseline, eligible participants are assigned in a 1:1 ratio to either ‘Access to biomarker’ or ‘No Access’ (week 0) through computer randomisation, stratified by sex and IBD subtype (Crohn’s disease versus ulcerative colitis) at trial enrolment. The randomisation will involve the use of random block sizes within each stratum.

The computer-generated randomised allocation sequence will be managed centrally, imported into the eCRF system and made available to site personnel. However, the allocation will not be accessible until the participant has signed the informed consent form and meets the eligibility criteria for study participation. Consequently, only authorised personnel will access information regarding the assignment of included patients, not future patients.

### Treatment arms

Patients will be stratified and follow the treatment strategy to which they have been randomly assigned. In the protein signature arm, patients with a protein profile indicating a heightened risk of an aggressive disease progression will undergo top-down treatment. In contrast, those with a protein signature indicative of an indolent disease course will be excluded from the trial. Patients randomised to the arm without access to the protein signature will receive treatment predicated on conventional clinical management. The top-down and clinical management arms are defined below.

#### Top-down treatment

Anti-TNF treatment, that is, infliximab or adalimumab, is started <2 weeks after stratification in participants randomised to access the protein profile and has a high-risk profile. The anti-TNF agent is paired with either azathioprine or 6-mercaptopurine, both of which are immunomodulators. All treatments should be prescribed at the discretion of the investigator. In contraindications, the investigator may opt against using immunomodulators.

#### Clinical management

The group randomised to the treatment arm with no access to the protein signature will be treated according to current medical practice, where study participants are treated using a ‘step-up’ pyramidal approach. Patients who fail first-line therapy undergo subsequent treatment escalation in a stepwise manner, as determined by the investigator.

### Discontinuation and participant withdrawal of subject

Participants can withdraw from the study without repercussions for their continued treatment. At any time, the investigator, sponsor or Data Safety Monitoring Board can remove a patient from the study. This may occur due to unacceptable side effects or non-compliance with study procedures. If a participant discontinues their involvement early in the study, follow-up will be done following an established clinical routine. Data collected until the withdrawal point may be included in the analysis. An ad hoc visit can be done to follow-up and terminate the participant’s participation in the study.

### Power and sample size calculation

The initial goal of the NORDTREAT study was to recruit 250 patients, focusing on those exhibiting a high-risk protein profile for IBD, based on a high-risk protein profile of 25% in the IBD Character cohort.^18^ However, an interim assessment in August 2023 (blinded to group allocation) suggested that the prevalence was approximately 17%. To achieve 85% power, a minimum of 26 participants is required in each high-risk group, assuming a remission rate of 0.75 in the ‘protein profile-driven top-down’ group and 0.35 in the clinical management step-up group at week 52. This calculation is based on Pearson’s χ^2^ two-group proportions test (two-sided, with continuity correction) at a significance level of 5%. Accordingly, 300 patients will be enrolled and divided into two groups of 150 patients each. One group will have access to protein profile information, while the other group will not. In both arms, 26 patients (17%) are expected to have a high-risk protein profile.

### Statistical methods, and procedures

The main analyses will be based on the intention-to-treat (ITT) population. The ITT principle asserts the effect of a treatment policy (ie, the planned treatment regimen) rather than the actual treatment given (ie, it is independent of treatment adherence). Hence, participants assigned to a treatment group (top-down treatment in predicted aggressive disease course patients) versus those treated according to clinical management will be followed up, assessed and analysed as members of that group, regardless of their adherence to the intended treatment plan (ie, independent of withdrawals and crossover phenomena). The primary outcome will be applied to the ITT population. Secondary outcome analysis will involve using multiple imputation techniques based on the Markov chain Monte Carlo method. Missing data are assumed to be missing at random. The robustness of analyses with missing data will be assessed through separate sensitivity analyses conducted on the ITT and per-protocol populations.

Dichotomous endpoints (including remission status and harms) will be analysed with logistic regression based on a generalised linear mixed model with the treatment group, centre, IBD condition and biomarker status (fixed effect) as covariates. Secondary outcomes will be compared using the same population and approach for statistical modelling as for the primary analyses, as far as they score proportions (of remission, response or AEs). Continuous outcomes will be analysed using repeated measures mixed linear models, incorporating the same fixed effects and using the baseline value of the relevant variable as a covariate. In addition to the principal secondary analysis mentioned earlier, which focuses on drug-related AEs in patients whose protein signature predicts a severe disease course in both randomisation groups, a safety analysis will also be conducted on all study participants (referred to as the safety population). This analysis will include reporting drug-related AEs for each study drug. On the publication of the results, the statistical codes employed in this trial will be made publicly available.

## Ethics and dissemination

### Ethics approval

Approval for the study was obtained from the Swedish Ethical Review Authority (Dnr 2020–03261) in Sweden, the Regional Ethics Committee south-east (reference number 180791) in Norway, the Danish Research Ethics Committees (Project-ID S-20200158) in Denmark, and the National Bioethics Committee (VSN-20–195) in Iceland. The procedures adhered to comply with the ethical standards of the responsible committee on human experimentation (institutional and national) and with the Helsinki Declaration of 1975 with later amendments.

On completion of the trial, the collected data will undergo a thorough analysis, tabulation and consolidation into a comprehensive final trial report. Once the analysis has been completed, the results will be shared with the scientific community through presentations at scientific conferences and submission for publication in a peer-reviewed journal. Press releases will be prepared to accompany publications to ensure that the trial’s findings reach a broader audience, including the global medical community, trial participants and patient organisations.

Authorship of the final trial outputs will strictly adhere to the guidelines set out by the International Committee of Medical Journal Editors. In preparing this article, we have followed the reporting guidelines outlined by the Standard Protocol Items: Recommendations for Interventional Trials.^19^

## supplementary material

10.1136/bmjopen-2023-083163 online supplemental file 1

## References

[1] Lirhus SS, Høivik ML, Moum B (2021). Incidence and prevalence of inflammatory bowel disease in Norway and the impact of different case definitions: A nationwide registry study. Clin Epidemiol.

[2] Agrawal M, Christensen HS, Bøgsted M (2022). The rising burden of inflammatory bowel disease in Denmark over two decades: A nationwide cohort study. Gastroenterology.

[3] Zhulina Y, Udumyan R, Henriksson I (2014). Temporal trends in non-stricturing and non-penetrating behaviour at diagnosis of crohn’s disease in Orebro, Sweden: a population-based retrospective study. J Crohn's Colitis.

[4] Burisch J, Pedersen N, Čuković-Čavka S (2014). East-west gradient in the incidence of inflammatory bowel disease in europe: the ECCO-epicom inception cohort. Gut.

[5] Feuerstein JD, Cheifetz AS (2014). Ulcerative colitis: epidemiology, diagnosis, and management. Mayo Clin Proc.

[6] Torres J, Mehandru S, Colombel J-F (2017). Crohn’s disease. Lancet.

[7] D’Haens G, Baert F, van Assche G (2008). Early combined immunosuppression or conventional management in patients with newly diagnosed crohn’s disease: an open randomised trial. Lancet.

[8] Khanna R, Bressler B, Levesque BG (2015). Early combined immunosuppression for the management of crohn’s disease (REACT): a cluster randomised controlled trial. Lancet.

[9] Lee JC, Lyons PA, McKinney EF (2011). Gene expression profiling of CD8+ T cells predicts prognosis in patients with crohn disease and ulcerative colitis. J Clin Invest.

[10] McKinney EF, Lee JC, Jayne DRW (2015). T-cell exhaustion, co-stimulation and clinical outcome in autoimmunity and infection. Nature New Biol.

[11] McKinney EF, Lyons PA, Carr EJ (2010). A CD8+ T cell transcription signature predicts prognosis in autoimmune disease. Nat Med.

[12] Biasci D, Lee JC, Noor NM (2019). A blood-based prognostic biomarker in IBD. Gut.

[13] Noor NM, Lee JC, Bond S (2024). A biomarker-stratified comparison of top-down versus accelerated step-up treatment strategies for patients with newly diagnosed crohn’s disease (PROFILE): A multicentre, open-label randomised controlled trial. Lancet Gastroenterol Hepatol.

[14] Vatn S, Carstens A, Kristoffersen AB (2020). Faecal microbiota signatures of IBD and their relation to diagnosis, disease phenotype, inflammation, treatment escalation and anti-TNF response in a european multicentre study (IBD-character). Scand J Gastroenterol.

[15] BIO IBD – A multi-modal national study to identify BIOmarkers for diagnosis, therapy response and disease progression in IBD.

[16] Fejrskov A, Füchtbauer JD, Davíðsdóttir LG (2024). Novel biomarker profiles to improve individual diagnosis and prognosis in patients with suspected inflammatory bowel disease: protocol for the nordic inception cohort study (NORDTREAT). BMJ Open.

[17] Maaser C, Sturm A, Vavricka SR (2019). ECCO-ESGAR guideline for diagnostic assessment in IBD part 1: initial diagnosis, monitoring of known IBD, detection of complications. J Crohns Colitis.

[18] Kalla R, Adams AT, Bergemalm D (2021). Serum proteomic profiling at diagnosis predicts clinical course, and need for intensification of treatment in inflammatory bowel disease. J Crohns Colitis.

[19] Chan A-W, Tetzlaff JM, Altman DG (2013). SPIRIT 2013 statement: defining standard protocol items for clinical trials. Ann Intern Med.

