# Supplementary material for: Nordic inflammatory bowel disease treatment strategy trial: protocol for the NORDTREAT randomised controlled biomarker-strategy trial
Source: BMJ Open. 2024 Jul 31;14(7):e083163. doi: 10.1136/bmjopen-2023-083163 (PMC11293405; doi:10.1136/bmjopen-2023-083163)
Supplement: online supplemental file 1 [file bmjopen-14-7-s001.pdf]

Information till forskningspersonerna

Vi vill fråga dig om du vill delta i forskningsstudien Nordisk studie av behandlingsstrategi vid inflammatorisk tarmsjukdom (NORDTREAT). I det här dokumentet får du information om studien och om vad det innebär att delta. Här finns också information om vad som förväntas av dig och om eventuella risker och fördelar med studien. Diskutera gärna studien med din läkare och ställ frågor om allt du undrar över innan du bestämmer om du vill vara med.

Vad är syftet med studien och varför blir jag tillfrågad om medverkan?

Inflammatorisk tarmsjukdom, IBD (på engelska Inflammatory Bowel Disease) är en sjukdom som kännetecknas av en kronisk inflammation i mag-tarmkanalen. Crohns sjukdom och ulcerös kolit är de två vanligaste formerna av inflammatorisk tarmsjukdom/IBD.

Du tillfrågas om du vill delta i delta i studien eftersom du utreds för eventuell inflammation i mag-tarm kanalen eller för att du nyligen har fått diagnosen IBD.

Sjukdomsförloppet vid IBD kan variera från mycket milda besvär till en aggressiv sjukdom som är svårbehandlad. I dag finns det inga markörer som på förhand kan ge information om det fortsatta sjukdomsförloppet för en enskild patient. Det är därför svårt att individanpassa behandlingen av IBD. I regel används en behandlingstrappa där man stegvis trappar upp behandlingen om man inte blir hjälpt av den inledande behandlingen.

De senaste tjugo åren har olika riktade terapier börjat användas vid behandling av IBD. Läkemedlen benämns biologiska läkemedel och består av antikroppar mot de ämnen som orsakar inflammation i tarmens slemhinna. Oftast sätts dessa mediciner in först när övriga läkemedel inte fungerar.

Forskning tyder på att det kan ge bättre resultat hos vissa patienter om man börjar med dessa biologiska läkemedel redan vid diagnos, innan sjukdomen hinner förvärras. Det är i dag mycket svårt att förutsäga vem som kommer utveckla en svår sjukdom och som därmed skulle ha nytta av tidig behandling med biologiska läkemedel. Det finns med andra ord ett stort behov av att tidigt kunna identifiera patienter som löper en högre risk för försämring

De senaste årens forskning har kunnat identifiera flera olika proteiner i blodet hos individer i samband med att de insjuknar i IBD. Dessa proteiner tror man kan förutsäga vilka IBD-patienter som kommer att behöva upptrappning av sin läkemedelsbehandling och som sannolikt skulle behöva en kraftfull läkemedelsbehandling redan från början.

Målet för denna studie är utvärdera om IBD-patienter som vid diagnos uppvisar dessa sjukdomsspecifika proteiner tjänar på att erhålla en kraftfull läkemedelsbehandling redan från början och samtidigt minska risken för att patienter med en mild form av sjukdomen får onödig läkemedelsbehandling.

Studien bedrivs vid ange lokalt sjukhus i samarbete med en nordisk forskargrupp (NORDTREAT) och kommer att inkludera totalt 300 patienter i Sverige, Norge, Danmark och Island. Forskningshuvudmän för studien är ange lokal forskningshuvuman (Region) här och Region Örebro län. Med forskningshuvudman menas den organisation som är ansvarig för studien. Studien är granskad och godkänd av Etikprövningsmyndigheten och Läkemedelsverket.

Hur går studien till?

Detta kommer att ske om du väljer att vara med i studien:

Vid samtliga besök kommer du att få träffa en läkare, berätta om hur du mår och gå igenom din läkemedelsbehandling samt lämna blodprov. Ditt deltagande i studien kommer att pågå i drygt ett år.

De flesta provtagningar och samtliga undersökningar som görs i studien är sådana som kommer att göras enligt rutin, även om du skulle välja att inte delta i studien.

Besök 1 (2-4 veckor innan besök 2)
Du kommer att bli tillfrågad om du vill delta i denna studie vid ett ordinarie besök som sker med anledning av att du utreds för, eller nyligen har diagnostiserats med, IBD. I samband med besöket finns det möjlighet att ställa frågor till sköterska och läkare som arbetar med studien. Om du vill delta i studien så får du lämna ditt samtycke till att inkluderas i studien med en underskrift innan någon undersökning för studien görs.

Om du samtycker till att delta i studien kommer du att få lämna blodprov. Förutom ett extra blodprov kommer du även att undersökas, få instruktioner för att lämna avföringsprover och besvara frågor om ditt välmående, vilka läkemedel du tar och din sjukdomshistoria. Samtliga frågor och undersökningar (undantaget ett av blodproven) kommer att genomföras även om du väljer att inte delta i studien då de är del av din planerade utredning. Om du väljer att delta i studien kommer du att få besvara vissa av frågeformulären online, vi behöver i så fall din e-post och mobiltelefon-nummer för att kunna skicka ut länken till frågeformuläret.

Det blodprov som tas särskilt för studien används för att mäta de proteiner som kan visa på om du har en ökad risk för en framtida försämring av din sjukdom. Du kommer att slumpvis hamna i en av två olika grupper i studien:

- För hälften av alla deltagare kommer denna protein-analys göras direkt och du och din läkare får svaret efter ungefär två veckor. Svaret avgör vilken behandling som sätts in. Om det tyder på att du har en ökad risk för att utveckla allvarlig sjukdom kommer du direkt att få behandling med ett biologiskt läkemedel, en så kallad TNF-hämmare. Tyder ditt provresultat på att du har en lägre risk för att utveckla en mer allvarlig sjukdom kommer du att behandlas enligt klinisk rutin, precis som om du inte deltagit i studien. Du kommer då inte att följas upp mer inom denna studie.

- För den andra gruppen kommer man inte att göra analysen av protein i blodet förrän efter studien avslutats, du som hamnar i denna grupp kommer att få den behandling du skulle ha fått om du inte deltagit i studien

Besök 2
Du kommer att få gå igenom en likadan läkarundersökning som vid första besöket och lämna blodprov igen. Vid detta besök kommer du även att få fylla i frågeformulär om ditt välmående och din sjukdom och läkemedel kommer att förskrivas utifrån vilken grupp du hamnat i

I samband med detta besök kommer en endoskopi av tjocktarmen att göras, en så kallad koloskopi. Vid en endoskopi används en böjlig slang med videokamera, med möjlighet att ta små vävnadsprover (biopsier) fråntarmslemhinnan. Några deltagare med symtom som tyder på Crohns sjukdom kommer också att behöva göra en undersökning av tunntarmen med magnetkamera. Såväl koloskopin som eventuell magnetröntgen av tunntarmen är undersökningar som görs som del i utredningen av dina besvär, även om du skulle välja att inte delta i studien.

Om du är kvinna i fertil ålder kommer du även få göra ett graviditetstest vid första besöket, då du inte bör vara gravid under behandling med vissa av de läkemedel som förskrivs i studien. Din läkare kommer att informera om riskerna vid de olika behandlingarna och vilka preventivmedelsmetoder som bör användas.

Besök 3 (12 veckor efter besök 2) och **Besök 4** (26 veckor efter besök 2)

Vid dessa besöks får du träffa en läkare för att gå igenom din läkemedelsbehandling och kontrollera hur du mår. Du får fylla i frågeformulär, lämna blodprov och får instruktioner för att lämna avföringsprover.

Besök 5 (52 veckor efter besök 2)

Som vid tidigare besök får du genomgå en läkarundersökning, gå igenom din läkemedelsbehandling, fylla i frågeformulär och lämna blod och avföringsprov. I samband med detta besök får du genomgå ännu en koloskopi då prover av tarmslemhinnan tas enligt normal rutin.

Möjliga följder och risker med att delta i studien

Beroende på vilken behandling din läkare rekommenderar för din IBD så kan du få olika biverkningar. Din läkare kommer att informera om vilken behandling du kommer att få och vilka biverkningar som just den kan orsaka och noga följa hur du mår under studien. Om du

mår dåligt eller känner att din hälsa på något sätt påverkas är det viktigt att du snarast informerar studieteamet.

Blodprovstagning kan vara lite obehaglig, ge blåmärken och i sällsynta fall kan en lokal inflammation uppstå. Vid varje blodprovstagning kommer 4 ml (knappt en tesked) blod att tas för studien, totalt 20 ml för samtliga besök (en dryg matsked), utöver de rutinprover du lämnar även om du inte deltar i studien.

Endoskopi görs i studien på samma sätt och med samma förberedelser som i vanlig rutin, vilket innebär att du får dricka laxermedel dagen innan för att tömma tjocktarmen på innehåll. Det gör att du måste ha omedelbar närhet till toalett vid dina förberedelser. Undersökningen är förenad med mer eller mindre obehag/smärta och i mycket sällsynta fall perforation (ett litet hål uppstår vid provtagning) av tarmen (1:5000), om detta sker behöver du övervakas på sjukhus under något dygn. Riskerna minimeras genom att undersökningen utförs av erfarna endoskopispecialister.

Vissa studiedeltagare med misstanke om Crohns sjukdom kommer att genomgå två undersökningar med magnetkamera. Observera att undersökningen inte ger någon röntgenstrålning och är densamma oavsett om du väljer att delta i studien eller inte

Det finns inga direkta fördelar för dig med att delta i studien, men resultaten från studien kan leda till bättre behandling för andra patienter med IBD i framtiden.

Vad händer med mina uppgifter?

Under studien kommer vi att samla in uppgifter om födelsedatum, kön, hälsodata (såsom nuvarande och tidigare sjukdomar) samt genomgångna undersökningar och behandlingar i studien. Insamlade uppgifter kommer att lagras i ett register och databehandlas. Ändamålet med detta register är forskning. Du har rätt att ta del av de uppgifter om dig som hanteras i studien, och vid behov få eventuella fel rättade. Du kan också begära att uppgifter om dig raderas samt att behandlingen av dina personuppgifter begränsas men då forskning betraktas som allmänt intresse så är det den rättsliga grunden för hantering av personuppgifter vilket innebär att forskningsdata inte alltid får raderas. Dina uppgifter är sekretesskyddade och ingen obehörig har tillgång till registret. Dina svar och dina resultat kommer att behandlas så att inte obehöriga kan ta del av dem. Vid databearbetning, då studien rapporteras eller publiceras, kommer enskilda individer inte att kunna urskiljas.

Enligt Dataskyddsförordningen, GDPR, (EU 2016/679) har du rätt att ansöka om information om vilka personuppgifter som behandlas och få rättelse av personuppgifter. Ange lokal huvudman (region) är ansvarig för behandlingen av personuppgifterna. Dataskyddsombudet är den person som ansvarar för att dina personuppgifter behandlas på ett lagligt och korrekt sätt. Vid behov kan dataskyddsombudet hjälpa till så att du kan få information om vad som registrerats och få eventuella rättelser genomförda. Dataskyddsombudet går att nå på: ange lokalt dataskyddsombud, adress, telefonnummer och mailadress.

Om du är missnöjd med hur dina personuppgifter behandlas har du rätt att ge in klagomål till Integritetsskyddsmyndigheten, som är tillsynsmyndighet (from 1 januari 2021 heter Datainspektionen istället Integritetsskyddsmyndigheten).

Dina personuppgifter kommer att användas för de ändamål som angivits ovan. De kan endast komma att behandlas för helt andra syften om du lämnat ett nytt samtycke och/eller nytt godkännande erhållits av Etikprövningsmyndigheten.

Förutom sjukvårdspersonal kan en person utsedd av studieansvarig (så kallad sponsor, i denna studie huvudansvarig forskare) eller en myndighetsperson komma att jämföra insamlad studiedata med din medicinska journal i kvalitetssyfte för att se om studien blivit rätt genomförd. Sponsors kvalitetsgranskare (monitor) måste underteckna en sekretessförbindelse innan de får tillgång till din medicinska journal. Studiedata sparas minst 10 år efter att studien är avslutad för att möjliggöra kontroller. Genom att du skriver under samtycket ger du din tillåtelse till denna insyn i din patientjournal.

Vad händer med mina prover?

Prover som tas i studien kommer att tillhöra en biobank i enlighet med Biobankslag (2023:38) som reglerar på vilket sätt prov får sparas och nyttjas. Biobankens namn är Ange aktuell biobank och den finns vid ange sjukhus . Huvudman (ansvarig) för biobanken är Ange lokal huvudman (Region) . Samtliga ovan nämnda prover kommer att vara kodade, vilket innebär att de inte kan kopplas direkt till dig som person. Kodnyckeln ansvarar forskningsansvarig läkare för och den förvaras oåtkomlig för obehöriga. Proverna kommer endast att användas för de ändamål som angivits ovan. De kan endast bli aktuella för analyser utöver IBD-forskning om du lämnat ett nytt samtycke och/eller nytt godkännande erhållits av Etikprövningsmyndigheten. Du kan, om du vill, redan nu signera ett samtycke till att sparade prover får användas till framtida forskning. Du har rätt att utan närmare förklaring begära att dina sparade prover skall förstöras.

Kodade prov kan komma att analyseras hos laboratorier eller andra samarbetspartners och företag inom EU, i Norge och på Island. Proverna får endast användas för de ändamål som du nu godkänner. Om prov som skickats till ett annat laboratorium finns kvar efter analys kommer det att förstöras.

Hur får jag information om resultatet av studien?

Resultaten kommer att publiceras i vetenskapliga tidskrifter och presenteras i samband med vetenskapliga möten. Data kommer att presenteras så att ingen enskild person kommer att kunna identifieras.

Du kan också be din läkare informera dig om studieresultatet när det finns tillgängligt.

Försäkring och ersättning

Att vara med i studien medför inga extra kostnader för dig. Rimliga resekostnader kommer att ersättas mot kvitto.

Liksom inom sjukvården i övrigt omfattas du av Läkemedelsförsäkringen och Patientskadeförsäkringen.

Deltagandet är frivilligt

Ditt deltagande är frivilligt och du kan när som helst välja att avbryta deltagandet. Om du väljer att inte delta eller vill avbryta ditt deltagande behöver du inte uppge varför, och det kommer inte heller att påverka din framtida vård eller behandling.

Om du vill avbryta ditt deltagande ska du kontakta den ansvariga för studien (se nedan).

Ansvariga för studien

Ansvarig läkare för studien är:

Ange lokalt ansvarig läkare, namn, e-mail, telefonnummer

Forskningssjuksköterska:

Ange namn, (e-mail,) telefonnummer

Samtycke till att delta i studien

Jag har fått muntlig och skriftlig informationen om studien *Nordisk studie av behandlingsstrategi vid inflammatorisk tarmsjukdom* och har haft möjlighet att ställa frågor. Jag får behålla den skriftliga informationen.

Jag bekräftar att:

- Jag ger mitt samtycke till att delta i studien och vet att mitt deltagande är helt frivilligt.
- Jag har tagit del av informationen om hur mina personuppgifter kommer att hanteras och att insamlad data om mig förvaras och hanteras elektroniskt av studieansvariga.
- Jag tillåter att utsedd monitor (kvalitetsgranskare) får ta del av mina patientjournaluppgifter som är relevanta för den aktuella forskningsstudien.
- Jag är medveten om att jag när som helst och utan förklaring kan dra tillbaka mitt samtycke och avsluta deltagandet.
- Jag ger mitt samtycke till att de prov jag lämnar kommer att sparas i biobank och att proven används för forskning i enlighet med vad som beskrivits i forskningspersoninformationen. Jag kan begära att få insamlade prov kasserade. Proven kommer då att förstöras alternativt avidentifieras.

Jag lämnar härmed mitt samtycke till att delta i studien:

Underskrift (forskningsperson) Namnförtydligande Datum

___________________________ ____________________________ ___________

Studieansvarig läkares underskrift

Jag har informerat om studien och forskningspersonen har fått tillfälle att ställa frågor.

Underskrift (läkare) Namnförtydligande Datum

___________________________ ____________________________ ___________

En kopia av denna signerade information ges till forskningspersonen.
