# Supplementary material for: Nordic inflammatory bowel disease treatment strategy trial: protocol for the NORDTREAT randomised controlled biomarker-strategy trial
Source: BMJ Open. 2024 Jul 31;14(7):e083163. doi: 10.1136/bmjopen-2023-083163 (PMC11293405; doi:10.1136/bmjopen-2023-083163)
Supplement: Supplementary file 2 [file bmjopen-2023-083163.reviewer_comments.pdf]

PEER REVIEW HISTORY

BMJ Open publishes all reviews undertaken for accepted manuscripts. Reviewers are asked to complete a checklist review form (**http://bmjopen.bmj.com/site/about/resources/checklist.pdf**) and are provided with free text boxes to elaborate on their assessment. These free text comments are reproduced below.

ARTICLE DETAILS

**TITLE (PROVISIONAL)**	The Nordic Inflammatory Bowel Disease Treatment Strategy Trial: Protocol for the NORDTREAT randomised controlled biomarker-strategy trial
**AUTHORS**	Rejler, Martin; Füchtbauer, Johannes David; Davíðsdóttir, Lóa; Fejrskov, Anja; Söderholm, Johan; Christensen, Robin; Andersen, Vibeke; Repsilber, Dirk; Kjeldsen, Jens; Høivik, Marte; Halfvarson, Jonas

VERSION 1 – REVIEW

**REVIEWER**	Barazesh, Mahdi<br>Gerash University of Medical Sciences
**REVIEW RETURNED**	21-Jan-2024

**GENERAL COMMENTS**	Dear authors your study protocol to somewhat describe comprehensive explanation of your designed study. Please also consider the following comments for more clarity.<br>- There are many typos and grammatical errors through the text that need to be edited by a native spoken English person.<br>- Please write the abbreviation of words for first use in text including MCS, EQ-5D, IBDQ, PCS, SF-36.<br>- Full name of some abbreviations is repeat twice for example: Intention to Treat (ITT).<br>- Some information and descriptions has been repeated several times in different sections of manuscript that need to be edited.<br>- The date of study design was not mentioned in this study protocol. Is this study currently ongoing?<br>- Discussion did not existed and only conclusions presented in the study protocol.<br>In protein profile what proteins were assayed as markers in patients with access to protein signature arm?<br>- Your statistical descriptions need more detail for clarity. Describe each statistical test in detail and their analysis outcomes.<br>_ please rewrite abstract to include more precise information.<br>- What parameter demonstrate by Each of IBDQ, the SF-36, and the EQ-5D outcome?<br>- - Please follow STROBE check list for including full patient information.<br>- Limitations of your study should be discussed in the text.

**REVIEWER**	Vasudevan, Abhinav<br>Monash University, Gastroenterology
**REVIEW RETURNED**	09-Apr-2024

**GENERAL COMMENTS**	The authors have provided the protocol for the NORDTREAT trial which is a multicentre randomised controlled trial stratifying

	treatment based on biomarkers for disease severity compared to standard care in IBD. This is a novel concept and an important innovation in personalised medicine for the treatment of IBD and is a commendable initiative. There are some aspects of the trial design that need further clarification as outlined below.<br><br>1. The use of both Crohn's disease and ulcerative colitis patients in the groups may lead to heterogeneity in the cohort. Can the authors provide an explanation of the choice to group them together rather than just limit it to one group? Is the biomarker stratification known to be equally effective in Crohn's disease and UC?<br><br>2. Please provide a list of permitted medications that are allowed in the cohort and what dosages are allowed (e.g. 5ASA therapy and corticosteroids).<br><br>3. It seems changes in therapy are allowed during the trial but these changes are at the discretion of the treating clinician. Can you please provide details regarding this. With the current treat to target paradigm that this mean that treatment optimisations and adjustments of doses of therapies are permitted? Are drug levels allowed. Please provide further details. Will medication changes result in exclusion from the intention to treat analysis?<br><br>4. Page 15, Top down strategy "The investigator may decide not to use immunomodulators for a subject if there are contraindications." Given the known benefit of combination anti-TNF therapy with immunomodulator therapy, should this be modified so that the investigator will use combination therapy unless there is a contraindication?<br><br>5. Page 16, Power and Sample size calculation The calculation seems to only be based upon high risk individuals rather than the whole cohort – can you please clarify who the primary end point will be evaluated between in the text? Is it only high risk individuals or all patients using the biomarker group versus not using it? Also, the combined clinical and endoscopic remission rate of 75% in the protein signature group seems to be quite high – please provide details of how this value was estimated.<br><br>6. Randomisation – please clarify who generates the randomisation sequence and whether these people are separate to the treating clinicians or any steps to conceal the allocation sequence from investigators<br><br>Minor points<br>Page 7, line 40 "treatment vs clinical" versus should be spelt in full<br>Page 7, line 47-58 "Time" should not be capitalised ("time") – similarly for the other secondary outcomes when separated by a comma rather than full stop<br>Page 14, line 46 "ID" should be spelled out in full when first used<br>Page 15, line 46 "immunomodulatory medications" should be "immunomodulators"

VERSION 1 – AUTHOR RESPONSE

Responses to Reviewer: 1 Dr. Mahdi Barazesh, Gerash University of Medical Sciences

Comments to the Author

Dear authors your study protocol to somewhat describe comprehensive explanation of your designed study. Please also consider the following comments for more clarity.

1. There are many typos and grammatical errors through the text that need to be edited by a native spoken English person.

Reply: Thank you for your detailed feedback. The revised manuscript has now been thoroughly edited by a professional, native-English-speaking proofreader.

2. Please write the abbreviation of words for first use in text including MCS, EQ-5D, IBDQ, PCS, SF-36.
- Full name of some abbreviations is repeat twice for example: Intention to Treat (ITT).
- Some information and descriptions has been repeated several times in different sections of manuscript that need to be edited.

Reply: We have now included the full expansion of all abbreviations at their first mention in the text, including the Mental Component Summary (MCS), EuroQol five-dimensions questionnaire (EQ-5D), Inflammatory Bowel Disease Questionnaire (IBDQ), Physical Component Summary (PCS), and 36-Item Short Form Survey (SF-36).

In addition, we have corrected the issue with the repeated expansion of Intention to Treat (ITT).

We have also carefully reviewed the manuscript for redundant information and descriptions across different sections and have streamlined the content to eliminate unnecessary repetitions.

3. The date of study design was not mentioned in this study protocol. Is this study currently ongoing?
- Discussion did not existed and only conclusions presented in the study protocol.

Reply: Yes, the study is ongoing, and information about the date for the inclusion of the first has been added to the paragraph on Trial sites, duration, and visits (page 10, lines 191-199) main document – marked copy).

“In February 2022, the trial started with the enrolment of the first patient.”

In accordance with the BMJ Open's author guidelines on Protocol papers, the manuscript does not include a Discussion section. Moreover, we have deleted the conclusion in the revised manuscript based on the Editor's recommendation (comment 1 above).

4. In protein profile what proteins were assayed as markers in patients with access to protein signature arm?

Reply: The proteins of the signature have not yet been disclosed. However, these data will be published as a separate full-length paper (manuscript in preparation) before the NORDTREAT trial is completed and data are analysed. This approach is similar to other biomarker discoveries, such as the PROFILE trial, where the protocol (PMID: 30523133)* and the biomarker signature (PMID: 31030191)** were published as two separate papers.

* Parkes, M., et al., *PRedicting Outcomes For Crohn's dIsease using a moLecular biomarkEr (PROFILE): protocol for a multicentre, randomised, biomarker-stratified trial.* BMJ Open, 2018. 8(12): p. e026767.

** Biasci, D., et al., *A blood-based prognostic biomarker in IBD.* Gut, 2019. 68(8): p. 1386-1395.

- Your statistical descriptions need more detail for clarity. Describe each statistical test in detail and their analysis outcomes.

Reply: The development of a complete statistical analysis plan (SAP) is ongoing and will be completed before the last patient has completed the trial i.e., before the study is unblinded. The section on Statistical methods, procedures and data analysis plan is based on the published protocol Study Details | The Nordic IBD Treatment Strategy Trial | ClinicalTrials.gov and EudraCT [2019-002942-19]. In our opinion, our statistical description is in line with BMJ Open's policies for protocol manuscripts. However, we are happy to expand the information about the statistical analyses if you and the Editor disagree.

- What parameter demonstrate by Each of IBDQ, the SF-36, and the EQ-5D outcome?

Reply: All three represent measures of quality of life and will be used for the analyses of "Other Secondary Outcomes, numbers 5-8" as outlined in Box 3 in the manuscript and inserted below.

5. The change from baseline in each of the 4 dimensions of the IBDQ at 52 weeks.

6. *Proportion of subjects with >20-point improvement from baseline in the IBDQ score at 52 weeks.*

7. *The change from baseline for each of the 8 individual subscales of the SF-36 and the PCS and MCS scores at 52 weeks*

8. *The changes from baseline in the EQ-5D dimensions, EQ-5D index, and health state VAS scores at 52 weeks.*

- Please follow STROBE check list for including full patient information.<br>- Limitations of your study should be discussed in the text.

Reply: We acknowledge the importance of the STROBE checklist for reporting epidemiological studies such as cohort, case-control, and cross-sectional studies. However, since this submission is a protocol manuscript, we have adhered to the SPIRIT checklist as per the author guidelines of BMJ Open. This ensures compliance with the specific reporting standards required for protocol papers.

In accordance with the author guidelines of BMJ Open, key limitations of the protocol are listed in the Article summary: Strengths and limitations of this study. As outlined in the response to comment 3, the manuscript does not include a Discussion section.

Responses to Reviewer: 2 Dr. Abhinav Vasudevan, Monash University

Comments to the Author

Comments to the Author:
The authors have provided the protocol for the NORDTREAT trial which is a multicentre randomised controlled trial stratifying treatment based on biomarkers for disease severity compared to standard care in IBD. This is a novel concept and an important innovation in personalised medicine for the treatment of IBD and is a commendable initiative. There are some aspects of the trial design that need further clarification as outlined below.

1. The use of both Crohn's disease and ulcerative colitis patients in the groups may lead to heterogeneity in the cohort. Can the authors provide an explanation of the choice to group them together rather than just limit it to one group? Is the biomarker stratification known to be equally effective in Crohn's disease and UC?

Reply: Thank you for your comment regarding the cohort composition. Our prognostic protein signature has been identified and validated in both Crohn's disease and ulcerative colitis patients. Additionally, our analyses have not revealed any significant interaction effects between these subtypes of IBD in the models used. Consequently, including both Crohn's disease and ulcerative colitis patients is justified and aligns with the study's objectives as outlined in the approved protocol Study Details | The Nordic IBD Treatment Strategy Trial | ClinicalTrials.gov and EudraCT [2019-002942-19]. The inclusion of both Crohn's disease and ulcerative colitis patients will also enhance the generalizability and applicability of our findings.

2. Please provide a list of permitted medications that are allowed in the cohort and what dosages are allowed (e.g. 5ASA therapy and corticosteroids).

Reply: Thank you for your inquiry about permitted medications and dosages. In the study arm without access to the protein signature, the protocol does not dictate specific drugs since treatment is based on conventional clinical management, allowing investigators discretion over medication choices. On the contrary, patients in the arm with access to the protein signature who display a protein profile associated with an increased risk of aggressive disease follow a top-down treatment algorithm. This includes the use of anti-TNF agents, with or without an immunomodulator. Additional details are provided in the approved protocol (section D: IMP), available at Study Details | The Nordic IBD Treatment Strategy Trial | ClinicalTrials.gov.

3. It seems changes in therapy are allowed during the trial but these changes are at the discretion of the treating clinician. Can you please provide details regarding this. With the current treat to target paradigm that this mean that treatment optimisations and adjustments of doses of therapies are permitted? Are drug levels allowed. Please provide further details. Will medication changes result in exclusion from the intention to treat analysis?

Reply: As outlined in the response to the above comment, specific drugs are not dictated by the protocol for the study arm without the protein signature, where treatment follows standard clinical management at the investigator's discretion. For patients in the protein signature arm identified with a

high-risk profile, dose adjustments of anti-TNF agents and immunomodulators are allowed under the top-down treatment algorithm. Furthermore, measuring drug levels is permitted. Importantly, changes in dosages will not exclude participants from the intention-to-treat analysis, aligning with the objectives of a biomarker-strategy study.

4. Page 15, Top down strategy “The investigator may decide not to use immunomodulators for a subject if there are contraindications.” Given the known benefit of combination anti-TNF therapy with immunomodulator therapy, should this be modified so that the investigator will use combination therapy unless there is a contraindication?

Reply: We acknowledge the efficacy of combination therapy involving anti-TNF and immunomodulators. However, the manuscript adheres to the protocol Study Details | The Nordic IBD Treatment Strategy Trial | ClinicalTrials.gov and EudraCT [2019-002942-19], which has been approved by the Ethical Review Authority and the Medical Product Agency in each participating country. As patient recruitment commenced in February 2022, it is not feasible to change the protocol at this stage.

5. Page 16, Power and Sample size calculation The calculation seems to only be based upon high risk individuals rather than the whole cohort – can you please clarify who the primary end point will be evaluated between in the text? Is it only high risk individuals or all patients using the biomarker group versus not using it? Also, the combined clinical and endoscopic remission rate of 75% in the protein signature group seems to be quite high – please provide details of how this value was estimated.

Reply: It is correct that our power and sample size calculation only target patients identified with a high-risk protein profile. (Patients with a low-risk profile in the arm with access to the protein signature are excluded from the trial.) This approach focuses our analysis on comparing high-risk patients in the protein signature group to the non-signature group.

The anticipated clinical and endoscopic remission rate of 75% in the protein signature group is derived from outcomes reported in the Step-Up versus Top-Down trial (PMID: 18295023)*** and the SEAVUE study (PMID: 35691323)****. Since these two trials only included patients with Crohn’s disease, we also utilised unpublished data on 1-year outcomes of both ulcerative colitis and Crohn’s disease patients in the IBSEN III cohort (personal communication with Principal Investigator Marte Lie Hoivik).

*** D'Haens, G., et al., *Early combined immunosuppression or conventional management in patients with newly diagnosed Crohn's disease: an open randomised trial.* Lancet, 2008. 371(9613): p. 660-667.

**** Sands, B.E., et al., *Ustekinumab versus adalimumab for induction and maintenance therapy in biologic-naive patients with moderately to severely active Crohn's disease: a multicentre, randomised, double-blind, parallel-group, phase 3b trial.* Lancet, 2022. 399(10342): p. 2200-2211.

6. Randomisation – please clarify who generates the randomisation sequence and whether these people are separate to the treating clinicians or any steps to conceal the allocation sequence from investigators

Reply: Thank you for raising this important point regarding randomization. The randomization sequence is generated by an independent statistical team using a computerized system, ensuring it remains separate from participating sites. Furthermore, to maintain the integrity of the study, the allocation sequence is concealed from all investigators using a centralized, secure web-based system. These measures prevent any potential bias and preserve the blinding of the study arms to both participants and treating clinicians until the point of assignment.

Minor points
Page 7, line 40 "treatment vs clinical" versus should be spelt in full
Page 7, line 47-58 "Time" should not be capitalised ("time") – similarly for the other secondary outcomes when separated by a comma rather than full stop
Page 14, line 46 "ID" should be spelled out in full when first used
Page 15, line 46 "immunomodulatory medications" should be "immunomodulators"

Reply: The typos have been corrected in the revised manuscript.

VERSION 2 – REVIEW

**REVIEWER**	Vasudevan, Abhinav<br>Monash University, Gastroenterology
**REVIEW RETURNED**	01-Jul-2024

**GENERAL COMMENTS**	The authors have addressed the suggested comments in the revised manuscript. I would suggest adding details of the allocation concealment to the manuscript and that the use of therapeutic drug monitoring and treatment optimisation is permitted in the study.<br><br>Minor point:<br>Page 9, line 181 - there is a duplicate full stop, please delete

VERSION 2 – AUTHOR RESPONSE

3. Reviewer: 2

The authors have addressed the suggested comments in the revised manuscript. I would suggest adding details of the allocation concealment to the manuscript and that the use of therapeutic drug monitoring and treatment optimisation is permitted in the study.

Reply: We appreciate the positive feedback on our previous revision. In response to the request, we have now extended the text regarding allocation concealment. As outlined, authorised personnel will only be able to access information regarding the assignment of included patients, not future patients.”

“The computer-generated randomised allocation sequence will be managed centrally, imported into the eCRF system and made available to site personnel. However, the allocation will not be accessible until the participant has signed the informed consent form and meets the eligibility criteria for study participation. Consequently, only authorised personnel will access information regarding the assignment of included patients, not future patients.”

In addition, we have clarified that therapeutic drug monitoring and treatment optimisation is permitted (page 9, lines 181-182).

“The use of therapeutic drug monitoring for treatment optimisation will be permitted.”

4. Minor point:

Page 9, line 181 - there is a duplicate full stop, please delete

Reply: Thank you for the thorough review, the typo has been corrected.
